# A novel integrated molecular and serological analysis method to predict new cases of leprosy amongst household contacts

**DOI:** 10.1371/journal.pntd.0007400

**Published:** 2019-06-10

**Authors:** Rafael Silva Gama, Márcio Luís Moreira de Souza, Euzenir Nunes Sarno, Milton Ozório de Moraes, Aline Gonçalves, Mariane M. A. Stefani, Raúl Marcel González Garcia, Lucia Alves de Oliveira Fraga

**Affiliations:** 1 Institute of Biological Sciences, Programa de Pós-Graduação em Ciências Biológicas-Imunologia e DIP/Genética e Biotecnologia, Universidade Federal de Juiz de Fora, Juiz de Fora, Minas Gerais, Brasil; 2 Núcleo de Pesquisa em Imunologia, Universidade Vale do Rio Doce / Univale, Governador Valadares, Minas Gerais, Brasil; 3 Basic Department of Health, Programa Multicêntrico de Bioquímica e Biologia Molecular, Universidade Federal de Juiz de Fora—Campus GV, Governador Valadares, Minas Gerais, Brasil; 4 Laboratório de Hanseníase, Fundação Oswaldo Cruz/ FIOCRUZ, Rio de Janeiro, Rio de Janeiro, Brasil; 5 Instituto de Patologia Tropical e Saúde Pública, Universidade Federal de Goías, Goiania, Goiás, Brasil; Institut Pasteur, FRANCE

## Abstract

**Background:**

Early detection of *Mycobacterium leprae* is a key strategy for disrupting the transmission chain of leprosy and preventing the potential onset of physical disabilities. Clinical diagnosis is essential, but some of the presented symptoms may go unnoticed, even by specialists. In areas of greater endemicity, serological and molecular tests have been performed and analyzed separately for the follow-up of household contacts, who are at high risk of developing the disease. The accuracy of these tests is still debated, and it is necessary to make them more reliable, especially for the identification of cases of leprosy between contacts. We proposed an integrated analysis of molecular and serological methods using artificial intelligence by the random forest (RF) algorithm to better diagnose and predict new cases of leprosy.

**Methods:**

The study was developed in Governador Valadares, Brazil, a hyperendemic region for leprosy. A longitudinal study was performed, including new cases diagnosed in 2011 and their respective household contacts, who were followed in 2011, 2012, and 2016. All contacts were diligently evaluated by clinicians from Reference Center for Endemic Diseases (CREDEN-PES) before being classified as asymptomatic. Samples of slit skin smears (SSS) from the earlobe of the patients and household contacts were collected for quantitative polymerase chain reaction (qPCR) of 16S rRNA, and peripheral blood samples were collected for ELISA assays to detect LID-1 and ND-O-LID.

**Results:**

The statistical analysis of the tests revealed sensitivity for anti-LID-1 (63.2%), anti-ND-O-LID (57.9%), qPCR SSS (36.8%), and smear microscopy (30.2%). However, the use of RF allowed for an expressive increase in sensitivity in the diagnosis of multibacillary leprosy (90.5%) and especially paucibacillary leprosy (70.6%). It is important to report that the specificity was 92.5%.

**Conclusion:**

The proposed model using RF allows for the diagnosis of leprosy with high sensitivity and specificity and the early identification of new cases among household contacts.

## Author summary

Leprosy is a chronic infectious disease caused by *Mycobacterium leprae* (*M*. *leprae*) that can infect cells in the skin and nerves. Despite efforts to eliminate leprosy, the number of *M*. *leprae* infected individuals who develop leprosy is still substantial in the world. The diagnosis relies mainly on clinical parameters. Histopathological and bacteriological analysis help to classify clinical forms of patients. Serology and polymerase chain reaction (PCR) assays are claimed by health professionals as auxiliary tools, but until now these tests have been used almost exclusively in research, with minor use in leprosy reference centers throughout Brazil. Here, we tested quantitative PCR (qPCR) designed to amplify specific *M*. *leprae* targets and ELISA assays to detect antibody response to recombinant antigens (LID-1, ND-O-LID). All results were analyzed by multivariate analysis based in artificial intelligence. We chose random forest as a classification algorithm to aid in the diagnosis and the monitoring of contacts. The results allowed us to diagnose cases of leprosy with high sensitivity and specificity and the early identification of new cases among household contacts.

## Introduction

Despite efforts to eliminate leprosy, the number of individuals infected with *Mycobacterium leprae (M*. *leprae*) who develop leprosy is still substantial, since the disease rate among the contacts of the person with leprosy should be considered. Some authors evaluate the efficiency of the decentralization of leprosy control through the contact examination. Still others point out that the routine practice of household contact examination is the most cost-effective approach to identifying new cases of leprosy [1].

Chemoprophylaxis and immunoprophylaxis have been evaluated as strategies for the control of leprosy. A randomized controlled study using chemoprophylaxis of a single dose of rifampicin (SDR) for contacts of patients showed a general protective effect of 60% in the first two years after the treatment. Vaccination with Calmette-Guérin (BCG) along with SDR had an additive protective effect, approaching 80% [2].

The importance of referral centers in supporting basic health services within the decentralization strategy has been highlighted, but the success of the program depends on the development of new tools to increase the accuracy of the diagnosis of leprosy [3]. Currently, serology and polymerase chain reaction (PCR) are claimed by health professionals as auxiliary tools. Until recently, these tests were mainly used in research, with minor use in leprosy reference centers throughout Brazil [4]. Several studies have used PCR to amplify specific sequences and target genes in *M*. *leprae* DNA, including repetitive sequences (RLEP) and the gene encoding the 16S subunit of ribosomal RNA (rRNA) [5–7]. Detection of *M*. *leprae* DNA in household contacts (HHC) is of high precedence, as these individuals have the highest risk of contracting the disease [8]. Thus, PCR has been evaluated by several research groups for use with this high-risk population [7, 9, 10].

In addition to the molecular assays, serological tests are utilized for detecting specific antibodies against *M*. *leprae* that indicate infection [11–18]. Serology results using *M*. *leprae* recombinant proteins commonly reflect the immunological spectrum of the disease [19]. In 2011, Sampaio et al. [20] identified three recombinant proteins (ML0405, ML2055, and ML2331) as immunogenic for leprosy. These three proteins were then fused together, generating a new compound called LID-1 [12, 21]. Subsequently, LID-1 and epitopes from the *M*. *leprae* phenolic glycolipid 1 (PGL-1) were combined to form ND-O-LID, assuring the immunoreactivity of the complex. The reactivity of the IgM and IgG antibody classes in human sera against to ND-O-LID was confirmed [22, 23].

A study conducted in a hyperendemic area of northern Brazil indicated LID-1 sensitivity for the diagnosis of leprosy as 89%, with a specificity of 42% [24]. Another study showed that ND-O-LID was able to detect a greater proportion of multibacillary (MB) and paucibacillary (PB) leprosy patients (87.0% and 32.3%, respectively) with 97.4% specificity. The ND-O-LID has a specificity of 85.89% and a sensitivity of 90.60% for MB leprosy and a mere 27.00% sensitivity for PB leprosy [25]. The analysis of antibody reactivity can also be used as a tool for early diagnosis of leprosy in HHC [9, 26–28]. It should be noted that IgM and IgG antibody reactivity against ND-O-LID allows for the detection of a significant number of infected individuals at an early stage of the disease [23]. Additionally, HHC that are seropositive for anti-LID-1 and anti-ND-O-LID present a higher risk of developing leprosy [12, 22, 25]. Both serological and molecular tests are used individually as diagnostic tools, notably for the MB clinical form, but the performance of such tests when analyzed independently is relatively low. Thus, it is imperative to promote an integrated analysis of these tests to obtain better parameters regarding the sensitivity and specificity for leprosy.

According to Baker et al. [29], multivariate methods provide an improved probability of detection over techniques based on single-parameter thresholds. Therefore, we chose the random forest (RF) algorithm, a multivariate statistical model based on artificial intelligence, for data analysis [30]. RF produces classification trees with minimum error rates that have the potential to aid clinical diagnosis and optimize public health service. RF was used in this study as an integrated analysis of molecular and serological methods to reach greater sensitivity and specificity in the diagnosis of MB leprosy and especially PB leprosy cases and to predict new cases of leprosy among HHC.

## Materials and methods

### Ethics statement

We hereby declare that this study was approved by the Ethics Committee of the Universidade Vale do Rio Doce (UNIVALE), filed under N° PQ 022/09-009. All participants signed a free and informed consent (IC) at the first evaluation. Parents/guardians provided consent on behalf of participants who were minors.

### Study group

The study was developed in Governador Valadares, Minas Gerais state, Brazil, a hyperendemic region for leprosy. This was a longitudinal study, including new cases diagnosed in 2011 and their respective HHC who were followed in 2011, 2012, and 2016. The study participants were patients at the Centro de Referência para Doenças Endêmicas e Programas Especiais (CREDEN-PES). New cases were classified according to regulations established by the Brazilian Ministry of Health that outlines the use of the Madrid classification and of operational classification for treatment purposes. All contacts were diligently evaluated by CREDEN-PES clinicians before being classified as asymptomatic. The endemic controls (EC) were recruited from a normal population and did not report having lived with patients with leprosy or having a family history of leprosy. They were clinically evaluated and did not present any other diseases.

**Fig 1:**
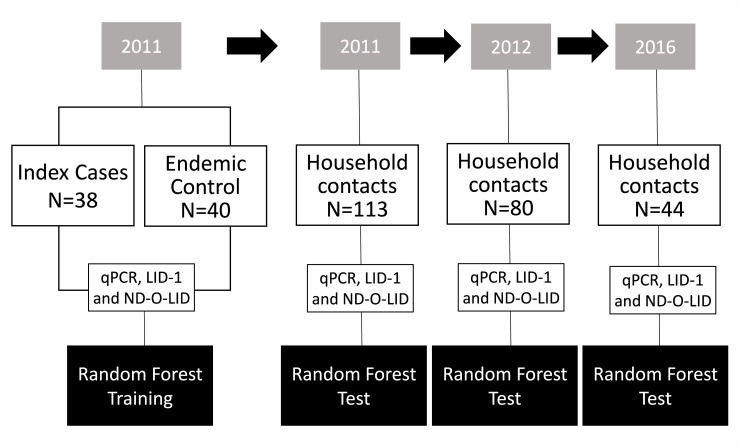
Flow chart of the follow-up study of household contacts (HHC). Biological samples from leprosy index cases and endemic controls clinically evaluated in 2011 were submitted to q-PCR and ELISA assays (anti-LID-1 and anti-ND-O-LID). Using data from these assays, the random forest algorithm was established for the prediction of a dichotomous model of Sick/Healthy. Then the same tests were applied to the HHC in 2011, 2012, and 2016. The random forest algorithm was appliedto predict Sick/Healthy individuals during the period of follow-up of HHC. q-PCR: quantitative polymerase chain reaction; ND-O-LID: natural disaccharide-octyl-leprosy IDRI diagnostic-1; LID-1: leprosy IDRI diagnostic-1.

In 2011, 196 subjects were admitted to the preliminary stage of this study (Fig 1). Of those subjects, 113 were HHC, 43 were new leprosy cases, and 40 were EC. The quantitative PCR (qPCR) results for all 43 leprosy cases were included in the analysis. However, due to inadequacies in some of the samples, the analysis for ELISA (anti-LID-1 and anti-ND-O-LID) included only 38 cases.

The cases were grouped as PB or MB. The PB group consisted of patients who presented with either undetermined or tuberculoid clinical forms, with a negative bacilloscopy. The MB group included patients clinically classified as dimorphous, with either negative or positive bascilloscopy, or virchowian, which presents a positive bascilloscopy. HHC were classified as household contacts of the PB group (HHCPB) or household contacts of the MB group (HHCMB). When examining the total number of HHC, there was a noted decrease from 2011 to 2012 and from 2012 to 2016 (113, 80, and 44 contacts, respectively). The reduction in the number of enrolled contacts may be due to address changes or the potential inability to return for additional clinical examinations or the collection of biological materials (blood and slit skin smears).

### Sample collection

Samples of slit skin smears (SSS) from the earlobe of the cases and HHC were collected according to the manual of smear microscopy in leprosy (BRASIL, 2010). In 2011 and 2012, SSS were collected only from the right earlobe. In 2016, SSS were collected from four sites (right and left ears, right and left elbows), increasing the chance of detecting a subclinical infection or confirming the absence of infection. The samples were stored in micro-centrifuge tubes containing 70% ethyl alcohol and kept in a freezer at -20°C until the extraction of DNA for the qPCR assay. Peripheral blood samples were collected for serological testing for the ELISA analysis. Data from blood qPCR analyses were not used in this study. The blood samples were used only for serological assays.

### DNA extraction and qPCR assay

DNA extraction was performed using the DNeasy Blood—Tissue kit (QIAGEN, Hilden, Germany) according to the manufacturer's specifications. DNA concentration in the eluted material was measured and analyzed using the NanoDrop 1000 spectrophotometer (Thermo Fisher Scientific, Waltham, MA, USA). qPCR was performed using the TaqMan qPCR amplification system (Thermo Fisher Scientific). The amplification target was the 16S rRNA gene region specific for *M*. *leprae*. The primer sequences and the probe sequence that were used in the assay were described previously [5] (S1 Table). The results of the qPCR are presented by the number of cycles (Ct) in which the accumulated fluorescence curve exceeded the cut line. As defined by the receiver operating characteristic (ROC) curve, Ct values smaller than 38.50 were considered positive. The value of Ct is inversely proportional to the amount of DNA present in the sample.

### Serological tests for antibodies

IgG anti-LID-1 (LID-1-lot: November 14, 2011, Dr. Duthie, USA) and IgG and IgM anti-ND-O-LID (ND-O-LID-lot: August 17, 2012, Dr. Duthie, USA) were detected by ELISA as described by Hungria et al. [31]. For anti-LID-1 and anti-ND-O-LID serology, the cutoff was determined by the ROC curve analysis using samples of leprosy cases and samples from EC. The serological test results were expressed as a mean absorbance of duplicates at 450 nm. Serum IgG and IgM levels of cases and EC, determined by ELISA, were compared by the Mann Whitney test, with a significance level of p ≤ 0.05.

### Statistical analysis

#### ROC curve

The ROC curve was employed for the ELISA and qPCR tests to analyze sensitivity, specificity, accuracy, likelihood ratio, and cutoff point associated with the least number of erroneous test results. The MedCalc Statistical program, version 5.00.020, was employed for this analysis. The ROC curve was established with data from all new cases diagnosed in 2011 and data from individuals in the EC group.

#### Integrated data analysis through random forest algorithm

The artificial intelligence-based classification model was applied using the RF package in the R program [32], a free software environment for statistical computing and graphics available at https://www.r-project.org [33]. RF is a combination of tree predictors, where each tree depends on the values of a random vector sampled independently and with the same distribution for all trees in the forest. The generalization error for forests converges asymptotically to a limit as the number of trees in the forest becomes large [30]. RF could predict the development of leprosy and clinical evolution of the disease by recognizing the pattern among the variables analyzed in the present study, with smallest classification error.

Three models for predicting the disease were evaluated: Madrid classification, operational classification, and dichotomous status (Sick/Healthy). For each of these models, a set of explanatory variables was used: age, gender, treatment time, qPCR (level of *M*. *leprae* DNA), serological level of IgG/IgM, and bacilloscopy index. Each decision tree of the RF models was obtained from the fit of 70% of the total number of leprosy cases and EC from the database. The remaining 30% of cases and EC were included to define a prediction. The prediction in RF was given by the majority vote of the classifiers (i.e. mode, as measure of central tendency).

A score was created based on the probability of correct prediction, i.e. prediction force (PF), which ranges from -1 to 1. This score is proportional to the program’s diagnostic accuracy. This means that the higher the score, the greater the likelihood of the individuals being Sick (PF = 1), while the lower the score, the greater the likelihood of the individuals being Healthy (PF = -1). The PF is represented as a bar graph.

The PF was calculated with the following equation:

$$PF=\left(\frac{2v}{t}\right)-1$$

where *v* = number of Sick votes and *t* = number of trees.

## Results

### Molecular Assay—qPCR

**Fig 2:**
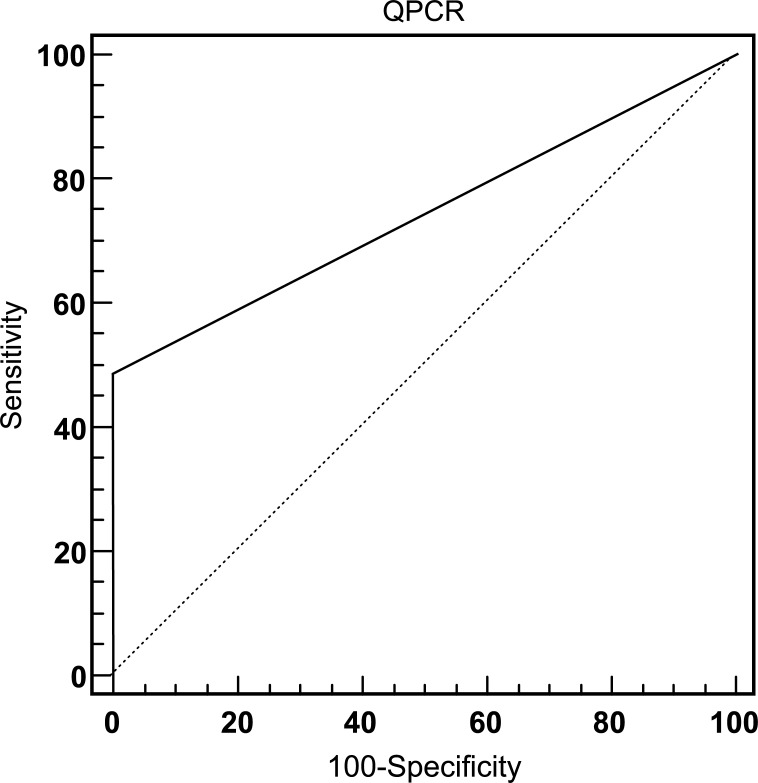
ROC curve for evaluation of qPCR performance in leprosy diagnosis. The ROC curve established the optimal cutoff point for qPCR. Sens: sensitivity; Spec: specificity; AUC: area under the curve; LR+: likelihood ratio positive; LR-: likelihood ratio negative.

The presence of *M*. *leprae* DNA in SSS of the earlobe collected in 2011 was evaluated by qPCR for 196 individuals, including 113 HHC, 43 cases of leprosy, and 40 individuals considered EC. The performance of qPCR in the diagnosis of leprosy cases was analyzed by means of the ROC curve (Fig 2). The cutoff value (38.5 Ct) was determined by the best relationship between sensitivity (48.8%) and specificity (100%) in the detection of *M*. *leprae* DNA among the 43 cases clinically diagnosed with leprosy (PB or MB) and 40 individuals considered EC. The area under the ROC curve had a value of 0.744, indicating diagnostic accuracy of the test.

The follow-up of HHC for the presence of *M*. *leprae* DNA was performed using SSS (qPCR SSS). We observed a reduction in the frequency of positive individuals during the study period. The frequency of positive HHCPB was 11.5% in 2011, 3.5% in 2012, and 0% in 2016. A similar reductive trend was noted among positive HHCMB: 19.7% in 2011, 9.5% in 2012, and 0% in 2016 (Table 1).

**Table 1:** Frequency of positive household contacts identified by qPCR SSS in 2011, 2012, and 2016.

Group	qPCR SSS	2011 N (%)	2012 N (%)	2016 N (%)
HHCPB	Positive	7 (11.5)	1 (3.5)	0 (0)
	Negative	45 (88.5)	28 (96.5)	25 (100)
**Total**		**52**	**29**	**25**
HHCMB	Positive	12 (19.7)	4 (9.5)	0 (0)
	Negative	49 (80.3)	38 (90.5)	21 (100)
**Total**		**61**	**42**	**21**

qPCR SSS: quantitative polymerase chain reaction of slit skin smears; HHCPB: household contacts of paucibacillary patients; HHCMB: household contacts of multibacillary patients.

### Serological assays

**Fig 3:**
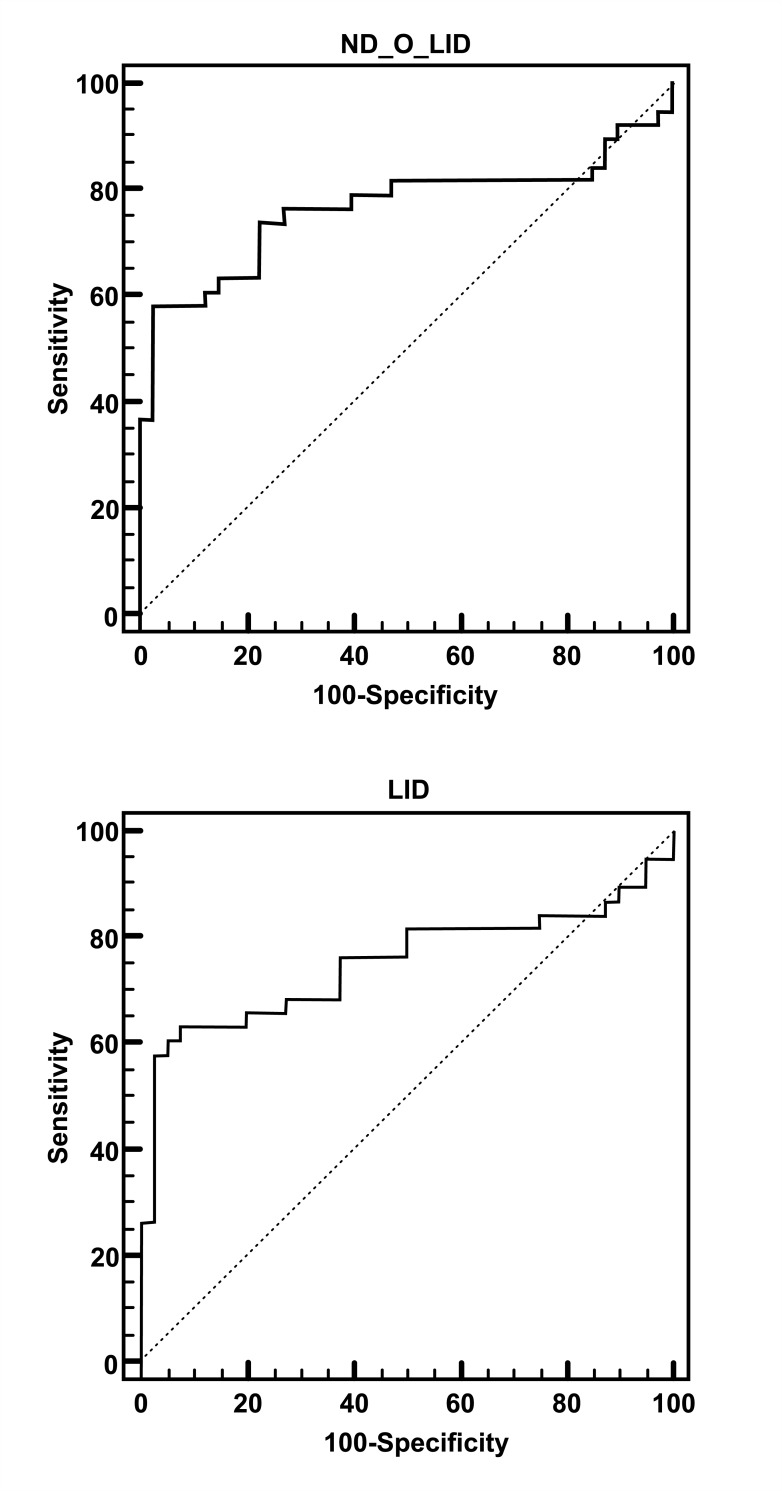
ROC curve analysis to evaluate ELISA assays. The ROC curve established the optimal cutoff point for ND-O-LID (A) and LID-1 (B). Sens: sensitivity; Spec: specificity; AUC: area under the curve; LR+: likelihood ratio positive; LR-: likelihood ratio negative.

The ELISA assays were conducted using the antigens ND-O-LID and LID-1, which have been shown to have good diagnostic properties for leprosy. To evaluate the performance of these recombinant antigens, the ROC curve was employed for 38 cases diagnosed in 2011 and 40 EC. In the anti-ND-O-LID ELISA test, a sensitivity of 57.9%, specificity of 97.5%, positive likelihood ratio of 23.16, negative likelihood of 0.43, and accuracy of 0.763 were obtained for detecting IgG and IgM antibodies using the cutoff of 1.0055. Concordantly, the anti-LID-1 ELISA test to detect IgG antibody, with a defining cutoff value of 0.4905, showed a sensitivity of 63.2%, specificity of 92.5%, positive likelihood ratio of 8.42, negative likelihood of 0.40, and an accuracy of 0.751 (Fig 3). No statistical difference was observed between the tests performed (p = 0.744).

**Fig 4:**
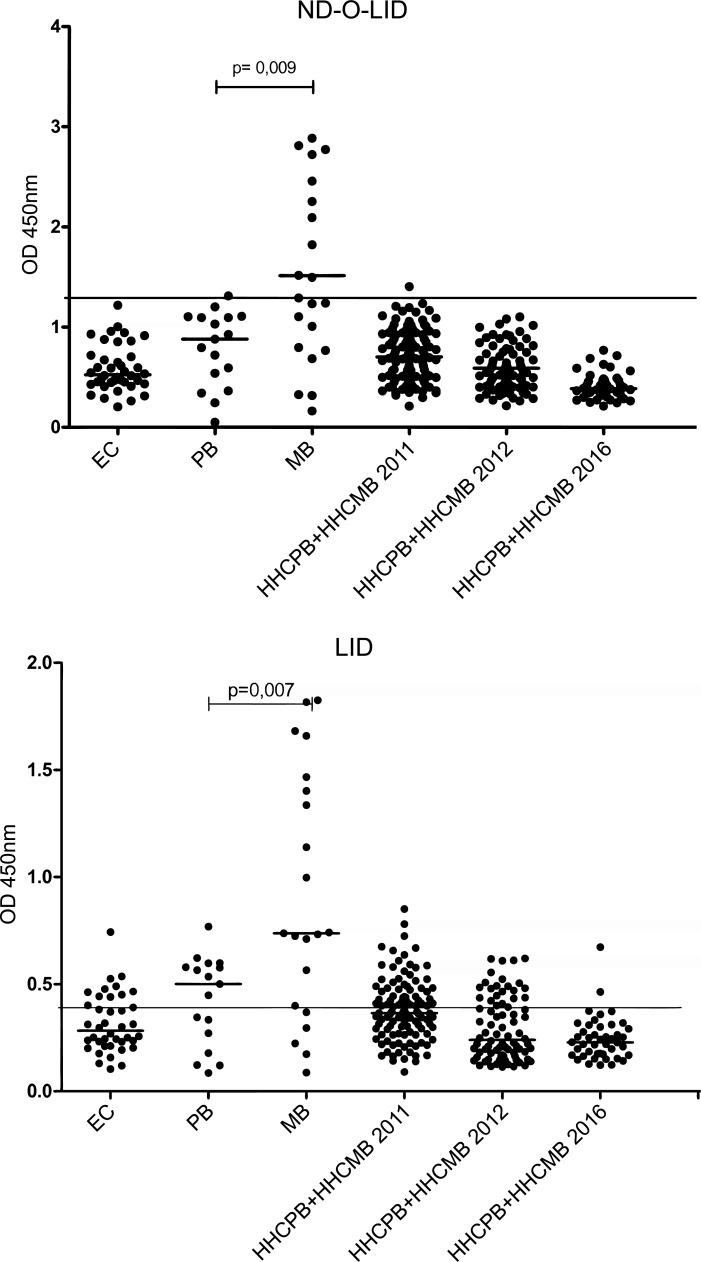
Comparison of the specific antibody responses in the EC, PB, MB, and HHC in 2011, 2012, and 2016 by ELISA assay. A) ND-O-LID; B) LID-1. EC: endemic control; PB: paucibacillary; MB: multibacillary; HHCPB: household contact of paucibacillary patient; HHCMB: household contact of multibacillary patient.

The levels of antibodies detected by the anti-ND-O-LID and anti-LID-1 ELISA assays were evaluated for the EC group, PB cases, MB cases, and HHC (Fig 4). Increased antibody production was observed for both ND-O-LID and LID-1 in the MB group. Similarly, the number of positive contacts identified by both tests decreased over the study period. These data corroborate with those of the qPCR analysis.

For the anti-ND-O-LID test, a positivity rate of 41.2% for PB and 71.4% for MB was identified among the leprosy cases. Conversely, the follow-up of HCC revealed a reduction in the positivity rate throughout the study (from 15.9% in 2011 to 0.0% in 2016). A similar profile was observed using the anti-LID-1 assay, with the frequency of positivity being 52.9% for PB and 71.4% for MB. The evaluation of HCC through the LID-1 assay also showed a reduction in positivity over the course of the study—18.6% in 2011, 11.2% in 2012, and 2.3% in 2016. It is interesting to note that the only individual (ID = 25) that showed a positive result in 2016 presented suspicious symptoms and in 2017 was diagnosed with leprosy by the health service (Table 2). Although the results in the tests highlight a higher sensitivity for anti-LID-1, we found that anti-ND-O-LID showed better indicators for specificity and positive likelihood ratio (Fig 2).

**Table 2:** Comparison of specific antibody responses in the EC, PB, MB and HHC groups in 2011, 2012, and 2016.

**Cases**	**Antigen**	**ND-O-LID**			**LID-1**		
	Year	2011					
	Group	EC n (%)	PB n (%)	MB n (%)	EC n (%)	PB n (%)	MB n (%)
	Positive	2 (5)	7 (41.2)	15 (71.4)	4 (10)	9 (52.9)	15 (71.4)
	Negative	38 (95)	10 (58.2)	6 (28.6)	36 (90)	8 (47.1)	6 (28.6)
	**Total**	**40**	**17**	**21**	**40**	**17**	**21**
**Household contacts**	**Antigen**	**ND-O-LID**			**LID-1**		
	Year	2011 n(%)	2012 n(%)	2016 n(%)	2011 n(%)	2012 n(%)	2016 n(%)
	Positive	18 (15.9)	4 (5)	0 (0)	21 (18.6)	9 (11.3)	1 (2.3)
	Negative	95 (84.1)	76 (95)	44 (100)	92 (81.4)	71 (88.7)	43 (97.7)
	**Total**	**113**	**80**	**44**	**113**	**80**	**44**

EC: endemic control; PB: paucibacillary; MB: multibacillary.

### Integration of molecular and serological tests by RF algorithm

The RF algorithm was used to integrate molecular and serological assays in the diagnosis of leprosy. One result of RF was the confusion matrix, which highlights the sensitivity and specificity. The models that use the Madrid classification and the operational classification present high specificity and low sensitivity (S2 Table and S3 Table). However, the model using the dichotomous classification (Sick/Healthy) presents better results of sensitivity (81.6%) and specificity (92.5%) (Table 3), and therefore, it was selected for the monitoring and the prediction of diagnosis among HHC.

**Table 3:** Confusion matrix for the dichotomous model (Sick/Healthy).

	Sick	Healthy	Sensitivity	Specificity
Sick	31	7	0,815	-
Healthy	3	37	-	0,925

### Variables defined for the dichotomous model (Sick/Healthy)

**Fig 5:**
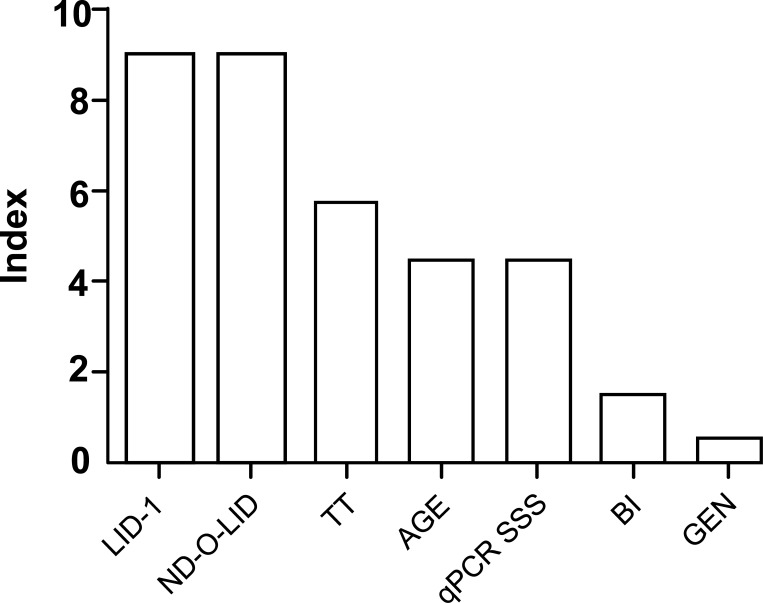
Levels of importance of the variables in the prediction model by random forest. TT: treatment time; qPCR SSS: quantitative PCR using samples of slit skin smears; BI: bacilloscopic index; GEN: gender.

The variables identified for the dichotomous model of Sick/Healthy were LID-1, ND-O-LID, treatment time, age, qPCR/SSS, bacilloscopic index, and gender. The importance level of each variable, based on an index of 0–10, was defined by RF (Fig 5).

### Error convergence curve according to the number of trees used in the random forest model

**Fig 6:**
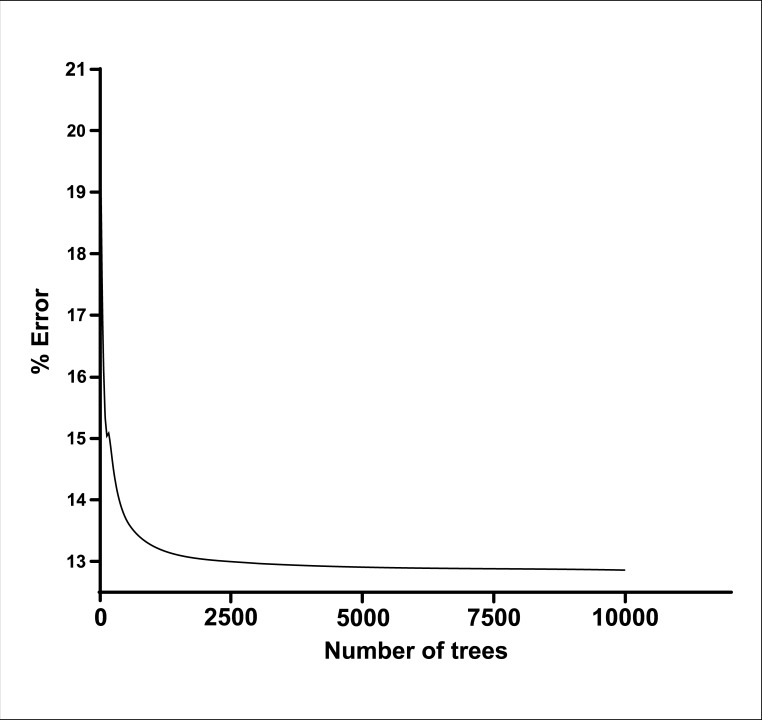
Error convergence curve according to the number of trees used in the random forest model.

Once the most suitable model was selected, the study proceeded with the optimization of the algorithm. The error convergence analysis was performed and defined the use of 10,000 decision trees with different error rates, establishing the error rate at 12.8% (Fig 6).

### Decision trees with their respective error rates

**Fig 7:**
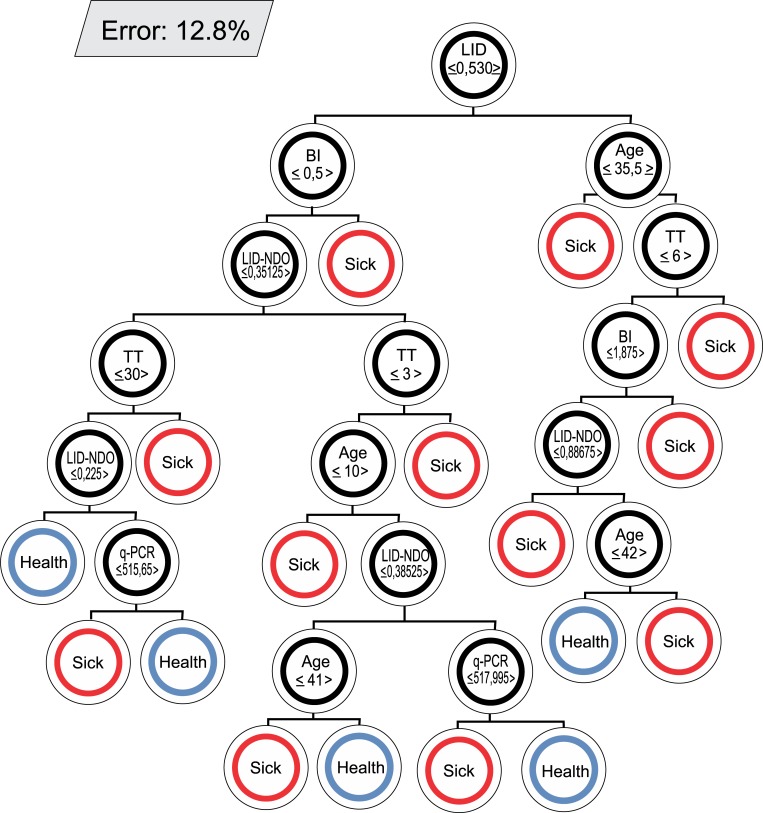
Example of a decision tree in the random forest model. Error 12.8%. BI: bacilloscopy index; TT: treatment time.

Fig 7, S1 Fig, and S2 Fig represent examples of decision trees included in the proposed model and their respective error rates. The pattern of each decision tree resulting from the training is unique. In each node divided into the tree, a binary decision based on the threshold of a variable value is imposed. The decisions imposed on each node are different for each tree. When no division occurs in a node, a diagnostic prediction is defined (Sick or Healthy).

### Frequency of positive results using various methods for diagnosis of PB and MB leprosy

Using the dichotomous model of Sick/Healthy, we compared the performance of RF in relation to bacilloscopy, qPCR/SSS, and ND-O-LID and LID-1 tests for the diagnosis of leprosy (Table 4). The model proposed in this study highlights the possible diagnosis of 90.5% of MB cases and 70.6% of PB cases. However, when isolated tests were evaluated, they did not reach the high diagnostic rate given by RF.

**Table 4:** Frequency of positive results using various methods for diagnosis of PB and MB leprosy.

Operational Classification	N	BacilloscopyN (%)	qPCR SSS N (%)	ND-O-LID N (%)	LID-1 N (%)	Random Forest N (%)
PB	17	0 (0.0)	3 (17.7)	7(41.2)	9(57.9)	12(70.6)
MB	21	11 (52.4)	11(52.4)	15(72.4)	15(72.4)	19(90.5)
Total	38	11 (28.92)	14 (36.8)	22(57.9)	24(63.2)	31(81.6)

N: number of individuals; qPCR SSS: qPCR slit skin smears; PB: paucibacillary; MB: multibacillary.

### Prediction Force (PF) defined by the dichotomous model (Sick/Healthy)

**Fig 8:**
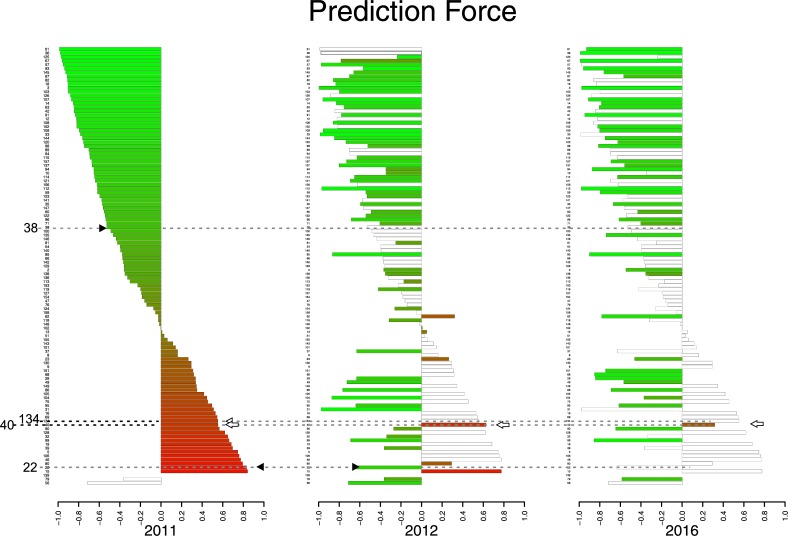
Prediction Force (PF) defined by the dichotomous model (Sick/Healthy). The bars represent each individual with the respective identification number. The height of the bar indicates the PF: low (PF ≤ 0.25), moderate (0.25 < PF ≤ 0.50), high (0.50 < PF≤0.75), and very high (PF > 0.75). The color scale emphasizes the PF—Healthy represented by green and Sick by red. The arrowhead indicates individuals (38, 134, 40, and 22 highlighted) who were clinically diagnosed as leprosy cases in 2012, and the white arrow shows an individual clinically diagnosed as a leprosy case in 2017. The white bars represent individuals who were lost during follow-up.

The PF of HHC was calculated in 2011, 2012, and 2016 for the monitoring and diagnosis of leprosy (Fig 8). In 2011, among the asymptomatic contacts classified as Sick, 48.6% had high or very high prediction strength. These data are suggestive of an increased risk of becoming ill, indicating a need to follow up on these individuals. In 2012, a reduction in the number of individuals with predicted sickness was observed, and only two individuals had a high or very high PF (Fig 8 arrowhead). In 2016, only one individual maintained the classification of Sick, with moderate PF. It is noteworthy that this individual (ID 40, indicated by white arrow in Fig 8) was correctly classified by RF as Sick at all three evaluation times of the study (2011, 2012, and 2016). In 2017, in the clinical evaluation, a histopathological examination confirmed the leprosy diagnosis for this individual. These data reinforce accurate predictions of the algorithm.

## Discussion

Early detection of *M*. *leprae* is a key strategy for disrupting the transmission chain of the disease and preventing the potential onset of physical disability. Clinical diagnosis is essential. However, some of the presented symptoms may go unnoticed, even by specialists [34]. In areas of greater endemicity, serological and molecular tests have been performed and analyzed separately for the follow-up of HHC, which are at high risk of developing the disease. The accuracy of these tests is still debated and it is necessary to make them more reliable, especially in the identification of cases of leprosy between HHC [10, 12, 17, 35–39]. We propose an integrated analysis of molecular and serological methods to better diagnosis and predict new cases of leprosy. Subsequently, it is important to supplement these test results using the before-mentioned RF. In this novel study, we obtained a higher sensitivity rate using RF for the diagnosis of new cases of leprosy, especially PB, making this an unprecedented analysis.

It is important to highlight the PCR findings (RLEP, Ag 85B, and 16S rRNA) with reasonable rates of sensitivity and specificity. The positivity of qPCR for SSS and/or blood samples may actually indicate the presence of bacillus or subclinical infection, although this does not mean that the condition will evolve into disease [4]. By analyzing the qPCR data separately, we confirmed their potential for detecting *M*. *leprae* DNA in cases of leprosy negative for SSS and in asymptomatic HHC. We found that 17.7% of PB and 52.4% of MB were positive by the qPCR-SSS. It is worth mentioning that among cases classified as MB, all dimorphous clinical forms with negative bacilloscopy were included (Table 4).

Generally, extensive evaluations of PCR tests in field studies have shown that the technique can reach 100% specificity, while sensitivity varies from 34% to 80% in PB and reaches more than 90% in MB [8]. Our data corroborate these studies, since we detected *M*. *leprae* infection with sensitivity and specificity rates of 48.8% and 100%, respectively (Fig 1).

As an invasive procedure, the collection of SSS (performed in 2011 and 2012) was limited to one specific site, the right ear lobe. Therefore, we believe that a higher frequency of positivity for qPCR could be achieved if additional collection sites were to be used, as is standard for smear microscopy [39].

Studies on leprosy transmission demonstrate that people living in proximity to leprosy cases are at increased risk of becoming ill [40, 41, 42]. Therefore, an effective strategy to reduce the incidence of leprosy is the monitoring of HHC and the diagnosis in the early stages of the disease. Banerjee et al. [35] demonstrated by multiplex PCR (M-PCR) a higher frequency of positivity in MB contacts (10.9%) than in PB contacts (1.3%). In our study, at the first time of evaluation (2011), a higher frequency of positive qPCR was found for HHCMB (19.7%) than for HHCPB (11.5%). The HHCMB are exposed to a higher bacterial load, which may have caused an increase in the frequency of qPCR positivity.

It is important to note that all HHC were considered clinically healthy. However, in our previous study working with the same group of HHC, we found 23.89% of contacts presented bacillary DNA, as evaluated by blood samples or SSS of the ear lobe [7]. This high rate of detection indicates high transmission dynamics of leprosy in the community. Knowing that leprosy has a long incubation period and the symptoms are difficult to detect in the early stages of the disease, we emphasize that the monitoring of HHC with positive results for qPCR is extremely relevant. In the first year of monitoring HHC, three new cases of leprosy were reported, indicating a co-prevalence rate of 2.65%. Of these three new cases, two individuals were of the HHCMB group and one was positive by qPCR before the onset of clinical symptoms.

Individuals who present positive serological results are considered to be infected by *M*. *leprae* [17]. However, this bacillus is known to have high infectivity and low pathogenicity; thus, the disease may not manifest in these individuals, even though they show antibodies specific for *M*. *leprae* antigens in their circulation [38]. On the other hand, according to Araújo et al. [9], high seropositivity in the population of an endemic area is worrisome, considering that individuals with subclinical infection may be a potential source of contamination for others. It is important to emphasize that a longitudinal study performed by Amorim et al. [16] showed that 3.6% of the HHC were diagnosed with leprosy, presenting positive results in the serological tests.

In 2011, using the cutoff obtained by the ROC curve, we identified 18.6% and 15.9% positive contacts for LID-1 and ND-O-LID, respectively. However, in 2016 we found a reduction in the positivity rate for LID-1 (2.3%) and ND-O-LID (0%). The application of LID-1 for the detection of cases up to one year before the recognition of lesions has been reported. However, this observation has not yet been formally demonstrated for ND-O-LID [43].

Frade et al. [17] showed that 62.8% of the patients clinically diagnosed with leprosy were positive by the ND-O-LID commercial rapid test (Orange Life, Rio de Janeiro, Brazil). However, this rapid test had lower specificity than the anti-PGL-1 and anti-LID-1 ELISA. According to Frade, although this test was able to identify dominant responses to both glycolipid (anti-PGL-1 IgM) and protein (anti-LID-1 IgG), ND-O-LID has the same limitations as other rapid tests for diagnosis, highlighting the difficulty of this test in monitoring individuals in the early stages of the disease and/or PB patients. Regardless of these findings, serological test results combined and associated with clinical examination may contribute to the early detection and treatment of leprosy cases.

In this study, we also observed a lower rate of positivity in the serology of cases in the PB clinical form. This can be explained by the predominance of the cellular immune response in these patients, to the detriment of the humoral response [44, 45].

Several reports indicated that substantial increases in titers for anti-LID-1 are important indicators of progression of leprosy, even in the absence of skin lesions or nerve damage [12, 46, 47]. In healthy subjects, high titers of anti-PGL-1 and anti-LID-1 suggest that there is an undetectable bacillary charge that stimulates the response to this antigen. For this reason, HHC that show substantial increases in anti-LID-1 and/or anti-PGL-1 titers should be monitored closely [27, 48].

A systematic and qualified approach to monitoring HHC is considered essential for disrupting the transmission of *M*. *leprae* [49]. Thus, we adopted a joint analysis of the serological and molecular tests, since the isolated analysis of these methods would not allow us to arrive at a reliable prediction. Multivariate methods provide an improved probability of detection over techniques based solely on single parameter thresholds [29]. The RF algorithm is unique because it is an innovative and robust analysis that allows for extremely accurate diagnostic predictions. We chose RF as a classification algorithm to aid in the diagnosis and the monitoring of HHC. Among the classification models studied, the one with the lowest mode error (12.8%) was obtained from the confusion matrix between Sick/Healthy, with a sensitivity of 81.58% and a specificity of 92.50%. We demonstrated that among the variables used in the selected dichotomous Sick/Healthy model, the serological tests (ELISA anti-LID-1 and anti-ND-O-LID) showed greater weight for the PF.

RF has been used to compare molecular signatures of several cutaneous diseases, including leprosy [50]. In that study, the authors obtained an overall error rate of 4.5% to differentiate leprosy, psoriasis, atopic dermatitis, or normal skin. The performance for each disease was assessed by sensitivity, specificity, and precision. Interestingly, in that study a patient was clinically diagnosed with atopic dermatitis, but the prediction of RF was for psoriasis. Later, the patient developed inflammatory plaques in the lower back, which were clinically diagnosed as psoriasis. In this way, the molecular classifier correctly predicted the diagnosis as the clinical course evolved.

In our study, the statistical analysis of the tests revealed sensitivity for anti-LID-1 (63.2%), anti-ND-O-LID (57.9%), qPCR SSS (36.8%), and smear microscopy (30.2%). However, the use of RF allowed for an impressive increase in sensitivity in the diagnosis of MB (90.5%) and especially PB (70.6%) (Table 4). It is important to report that the specificity was 92.5% for both (Table 3).

Upon comparing recent data from the literature [17, 51] we found different sensitivity and specificity rates for the applied serological tests, ranging from 88% sensitivity (anti-LID-1 ELISA) for MB to the extremely low value of 6% (anti-ND-O-LID ELISA) for PB [38]. Thus, the methodology used in our study contributes to a more rigorous assessment of the performance of the tests and assists in the diagnosis of leprosy, mainly in the PB clinical form, in which the antibody production and the bacillary load are characteristically reduced.

We evaluated the use of RF in the monitoring of HHC over the span of five years. We found a high index of contacts with prediction of "Sick" in the year 2011. Although these individuals did not present enough clinical symptoms to conclude the diagnosis of leprosy, our data indicated that they should be submitted to a periodic clinical evaluation, since the prediction "Sick" suggests subclinical infection and indicates that the individual will more than likely become ill. This information was confirmed in 2012 with the clinical diagnosis of three new cases among the HHC. There was also a significant reduction in the frequency of "Sick" prediction in 2012, as expected (Fig 8). This fact may be related to decreased exposure to bacillus after treatment of the case and to activation of the immune system by BCG administration [52]. In 2016, only one individual continued with the "Sick" prediction first established in 2011. It is noteworthy that in 2017 this individual (ID = 40) was clinically re-evaluated and his histopathological examination confirmed the diagnosis of dimorphous (MB) leprosy. Surprisingly, we also observed a great change in the frequency of prediction "Sick" to "Healthy." Notably, we found that in HHC the number of "Sick" predictions was reduced over the follow-up, reinforcing the correct classification and indicating the evolution of subclinical infection to being cured (Fig 8).

Although the modeling did not consider the data missing (NAs) from the study, there are ways to deal with this bias. In the random forest algorithm, available for free in the R program, there is a function called “rfImpute” that starts by imputing NAs using medians or modes. Then “random forest” is called with the completed data. The proximity matrix from the random forest is used to update the imputation of the NAs. For continuous predictors, the imputed value is the weighted average of the non-missing observations, where the weights are the proximities. For categorical predictors, the imputed value is the category with the largest average proximity. This process is iterated several times. Therefore, it would be possible to predict the probability of a patient being ill, even if the patient lacks a certain data collection from the longitudinal study, given the context of the parameters of the group being evaluated.

Individual ID-38 was considered healthy in 2011, both by physicians (clinically) and by the prediction model we adopted (RF). However, this individual was clinically diagnosed as sick in 2012 but did not have the immunological and molecular tests performed, so he could not be evaluated by our prediction model that same year.

Individual ID-22 was considered clinically healthy in 2011 but was classified as Sick by RF. In 2012, this individual was diagnosed as sick (clinically) by physicians but classified as Healthy by RF, indicating the apparent inconsistency. However, RF considers immunological and molecular tests, unlike physicians who use exclusively physical resources for their clinical conclusions. We could consider that the RF anticipated in 2011 the clinical diagnosis of 2012 and that the individual, in that year, could be presenting immunological and molecular characteristics that approached the healthy profile. Another hypothesis (which lacks proof) would be that the immune system of this individual was able to reduce the bacillary load present in his body in the previous year.

It is important to note that our prediction model has the potential to be applied in the Vale do Rio Doce region, which in 2018 recorded about 1% of all cases of leprosy in Brazil [53]. We do not, however, rule out the possibility of expanding this study to national and international levels. The purpose of this expansion would be to bring to light scientific knowledge and promising methodologies for the eradication of leprosy, as greatly encouraged by WHO [54]. A more comprehensive evaluation of the integrated use of serological and molecular tests in the early diagnosis of leprosy through random forest becomes necessary, since it could aid in more accurately interrupting the *M*. *leprae* transmission chain. Therefore, we suggest a randomized multicenter study for validation of the model.

It is also important to consider the loss of participants from the study over time as a study limitation. This loss was due to several reasons, including prolonged follow-up (which may imply the change of address of the participant), the stigma related to illness that makes direct access to the patient's residence difficult, and the potential inability to return for additional clinical examination or for the collection of biological materials (blood and SSS).

Many leprosy-endemic countries would find it difficult to perform all the molecular and serological tests to predict the development of leprosy in HHC. However, a rapid and low-cost molecular loop-mediated isothermal amplification (LAMP) has been recommended by the WHO for the diagnosis of pulmonary tuberculosis [55]. Likewise, we believe that molecular and serological assays could be implemented in reference laboratories and the RF analysis model be applied to predict new cases of leprosy among HHC. Those who presented positive results would be referred for chemoprophylactic treatment and immunoprophylaxis (BCG). In this context, our model could contribute to leprosy control strategies, especially early diagnosis and referral to chemoprophylaxis in a safe way.

We conclude that the model proposed by the RF allows the diagnosis of cases of leprosy with high sensitivity and specificity and the early identification of new cases among HHC. In addition, our novel study allows for the targeting of chemoprophylaxis exclusively for those predicted to have subclinical infection, contributing to the effective control of leprosy.

## Supporting information

S1 Table Sequence of primers and probe used in q-PCR. (DOCX)

S2 Table Confusion matrix for Madrid classification model. UND = Undetermined; TB = Tuberculoid; DM = Dimorphous; VV = Virchowian
(DOCX)

S3 Table Confusion matrix for operational classification model—PB x MB. MB = Multibacillary; PB = Paucibacillary
(DOCX)

S4 Table Demographic characteristics of study groups. RF: random forest; Min: minimum; Max: maximum; n: number of individuals; TT: treatment time; BI: bacilloscopy index; NA: not applicable
(DOCX)

S1 Fig Example of decision tree in the random forest. Error 15.1%. BI: bacilloscopy index; TT: treatment time.
(EPS)

S2 Fig Example of decision tree in the random forest. Error 20%. BI: bacilloscopy index; TT: treatment time.
(EPS)
