# Supplementary material for: A novel integrated molecular and serological analysis method to predict new cases of leprosy amongst household contacts
Source: PLoS Negl Trop Dis. 2019 Jun 10;13(6):e0007400. doi: 10.1371/journal.pntd.0007400 (PMC6586366; doi:10.1371/journal.pntd.0007400)
Supplement: S1 Table — (DOCX) [file pntd.0007400.s001.docx]

| **Gen** | **Description** | ***Primer* / Probe** | **Sequence** |
| --- | --- | --- | --- |
| 16S rRNA | 16S rRNA | ML16S rRNATaq-F | 5`-GCA TGT CTT GTG GTG GAA AGC-3` |
|  |  | ML16S rRNATaq-F | 5`-CAC CCC ACC AAC AAG CTG AT-3` |
|  |  | ML16SrRNATaq-Probe | 5`-CAT CCT GCA CCG CA-3` |
