# Supplementary material for: A novel integrated molecular and serological analysis method to predict new cases of leprosy amongst household contacts
Source: PLoS Negl Trop Dis. 2019 Jun 10;13(6):e0007400. doi: 10.1371/journal.pntd.0007400 (PMC6586366; doi:10.1371/journal.pntd.0007400)
Supplement: S2 Table — UND = Undetermined; TB = Tuberculoid; DM = Dimorphous; VV = Virchowian (DOCX) [file pntd.0007400.s002.docx]

|  | UND | TB | DM | VV | HEALTH | SENSITIVITY | SPECIFICITY |
| --- | --- | --- | --- | --- | --- | --- | --- |
| UND | 0 | 1 | 1 | 0 | 1 | 0 | - |
| TB | 0 | 3 | 4 | 0 | 7 | 0,21 | - |
| DM | 1 | 2 | 7 | 1 | 3 | 0,50 | - |
| VV | 0 | 0 | 2 | 5 | 0 | 0,71 | - |
| HEALTH | 0 | 3 | 0 | 0 | 37 | - | 0,92 |
