# Supplementary material for: A novel integrated molecular and serological analysis method to predict new cases of leprosy amongst household contacts
Source: PLoS Negl Trop Dis. 2019 Jun 10;13(6):e0007400. doi: 10.1371/journal.pntd.0007400 (PMC6586366; doi:10.1371/journal.pntd.0007400)
Supplement: S3 Table — MB = Multibacillary; PB = Paucibacillary (DOCX) [file pntd.0007400.s003.docx]

|  | MB | PB | HEALTH | SENSITIVITY | SPECIFICITY |
| --- | --- | --- | --- | --- | --- |
| MB | 14 | 5 | 2 | 0.67 | - |
| PB | 5 | 5 | 7 | 0.29 | - |
| HEALTH | 0 | 2 | 38 | - | 0.95 |
