# Supplementary material for: A novel integrated molecular and serological analysis method to predict new cases of leprosy amongst household contacts
Source: PLoS Negl Trop Dis. 2019 Jun 10;13(6):e0007400. doi: 10.1371/journal.pntd.0007400 (PMC6586366; doi:10.1371/journal.pntd.0007400)
Supplement: S4 Table — RF: random forest; Min: minimum; Max: maximum; n: number of individuals; TT: treatment time; BI: bacilloscopy index; NA: not applicable (DOCX) [file pntd.0007400.s004.docx]

| **RF – Training** | **RF - Test** |
| --- | --- |
| **Group:** | **Group:** |
| Index Cases and Endemic Control | Household Contacts |
| **Age (years)** | **Age (years)** |
| Min: 8  Max: 92  Mediana: 37 | Min: 5  Max: 92  Mediana: 30 |
| **Gender (n)** | **Gender (n)** |
| Female - 40  Male - 38 | Female - 66  Male - 47 |
| **TT (days)** | **TT (days)** |
| Min.: 0  Max.: 150 | NA |
| **BI** | **BI** |
| Min.: 0  Max.: 4,25 | NA |
